# Cervical cancer screening among English- and Spanish-speaking Hispanic women in an urban safety net health system, 2015–2020

**DOI:** 10.1186/s12905-023-02448-3

**Published:** 2023-06-15

**Authors:** Trisha L. Amboree, Susan Lackey Parker, Shaun Bulsara, Matthew L. Anderson, Kathleen M. Schmeler, Elizabeth Y. Chiao, Jane R. Montealegre

**Affiliations:** 1 Department of Behavioral Science, The University of Texas MD Anderson Cancer Center, 1515 Holcombe Blvd., Unit 1330, Houston, TX 77030 USA; 2 Department of Pediatrics, Baylor College of Medicine, Houston, TX USA; 3 Dan L. Duncan Comprehensive Cancer Center, Baylor College of Medicine, Houston, TX USA; 4 Division of Gynecologic Oncology, University of South Florida Morsani School of Medicine and Tampa General Hospital Cancer Institute, Tampa, FL USA; 5 Department of Gynecologic Oncology and Reproductive Medicine, The University of Texas MD Anderson Cancer Center, Houston, TX USA; 6 Departments of Epidemiology and Medical Oncology, The University of Texas MD Anderson Cancer Center, Houston, TX USA

**Keywords:** Cervical cancer prevention, Nativity, Hispanic populations, Disaggregation

## Abstract

**Background:**

The Hispanic population is heterogeneous with differences in health behaviors across subgroups by nativity and preferred language. We evaluated cervical cancer screening adherence among English- and Spanish-speaking Hispanic patients receiving care at a safety net health system.

**Methods:**

Electronic health records were used to identify 46,094 women aged 30–65. Up to date (UTD) screening was defined based on date of last Pap test, human papillomavirus (HPV) test, or Pap/HPV co-test.

**Results:**

Overall, 81.5% of 31,297 Hispanic women were UTD. English-speaking Hispanic women had a lower prevalence of being UTD when compared to Spanish-speaking Hispanic women (aPR: 0.94, 95% CI: 0.93 – 0.96). Further, those with indigent healthcare plans had a higher prevalence of being UTD when compared to those with private insurance (aPR: 1.10, 95% CI: 1.09 – 1.12), while all other health insurance plans were associated with lower UTD screening when compared to private insurance.

**Conclusions:**

These findings suggest screening differences within the Hispanic population, highlighting the need for disaggregated research assessing heterogeneity within racial/ethnic groups, specifically among Hispanic populations.

**Supplementary Information:**

The online version contains supplementary material available at 10.1186/s12905-023-02448-3.

## Background

Cervical cancer is almost completely preventable through vaccination against high-risk genotypes of the human papillomavirus (hrHPV), adherence to routine screening, and treatment of pre-cancerous lesions [1]. Current cervical cancer screening guidelines reflect the fact that population-based estimates for the prevalence of active hrHPV infections decline with age [2]. In 2018, the United States Preventive Services Task Force (USPSTF) recommended a) for women aged 21 to 29 years to be screened by cytology (i.e., Papanicolaou or Pap test) every 3 years [2]; and b) for women aged 30–65 years to be screened by Pap testing alone every 3 years or hrHPV testing alone or in combination with Pap testing every 5 years [2]. After age 65, USPSTF recommendations allow low-risk women to discontinue screening [2]. Unfortunately, only an estimated 77% of women in the United States (U.S.) are up to date (UTD) on screening according to these guidelines [3], which is below the Healthy People 2030 goal of 84.3% [4]. This contributes to over 14,000 cervical cancer diagnoses and 4,000 cervical cancer deaths each year in the U.S. [1].

In the U.S., there are substantial racial/ethnic disparities in screening access and utilization, leading to significant disparities in cervical cancer burden. Racial/ethnic minorities are recognized as minoritized groups that have been subject to structural racism and inequity compared to the non-Hispanic White majority population. Women of Hispanic/Latino ethnicity (henceforth termed Hispanic) are the racial/ethnic minority group with the highest incidence of cervical cancer, with an age-adjusted incidence of 9.7 per 100,000 compared to 7.0, 8.4, 9.0, and 5.7 among women of non-Hispanic White, non-Hispanic Black, non-Hispanic American Indian/Alaska Native, and non-Hispanic Asian/Pacific Islander race/ethnicity, respectively [5]. Nationally, Hispanic women are less likely to be UTD with cervical cancer screening compared to their non-Hispanic White counterparts. While Hispanic women are often considered a single group in the literature, studies show that there are significant differences in cervical cancer outcomes and preventive behaviors within the Hispanic population. For example, foreign-born (i.e., immigrant) Hispanic women are 10% more likely to be diagnosed with cervical cancer at a late stage [6] and are significantly less likely to be UTD on screening. This may be because immigrants face multiple challenges gaining access to care [7], including lack of healthcare insurance, which is believed to be the main reason for their reduced prevalence of UTD screening. In the U.S., health insurance is predominantly through commercial plans which are sponsored by employers or paid for directly by individuals (termed private insurance). A recent national study found that only 56% of screening-eligible foreign-born Hispanic women had health insurance, compared to 81% among their U.S.-born counterparts [8].

Disaggregated analyses by nativity are often not possible because place of birth is generally not available in the electronic health record (EHR). However, preferred language is often collected and can be used as a surrogate for immigrant status. In the U.S., 54.6% of foreign-born Hispanics report Spanish as their preferred/primary language, compared to 5.3% of U.S.-born Hispanics [9]. In the general population, English-speaking Hispanic women are known to have a higher rate of UTD cervical cancer screening compared to Spanish speakers [10]. However, it is unknown whether reduced odds of screening among immigrant women persists in women who have access to healthcare and screening, as is the case of patients within some publicly funded “safety net” health systems. In the absence of universal healthcare in the U.S., safety net health systems are those that provide healthcare to uninsured patients, patients who have publicly funded healthcare coverage (termed Medicaid), and other vulnerable populations [11].

This paper aims to assess cervical cancer screening adherence among English- and Spanish-speaking Hispanic women who have access to healthcare through a publicly funded, urban safety net health system. In doing so, it aims to highlight the importance of disaggregating data in heterogenous populations to elucidate disparities that may not be apparent when looking at the population as a whole [12, 13].

## Methods

### Setting

Harris County, TX (population approximately 4.8 million) and its county seat, Houston, is one of the most diverse counties in the U.S. The population is 44% Hispanic, 28% non-Hispanic White, 20% Black or African American regardless of ethnicity, and 11% Asian, American Indian or Alaska Native, or Native Hawaiian or Pacific Islander regardless of ethnicity [14]. Immigrants comprise 26% of the population [14] with an estimated 587,000 (37%) undocumented immigrants living in the Houston metropolitan area (i.e., living in the U.S. without legal authorization) [15]. The county has one of the highest proportions of uninsured adults (22%) [14]. Harris County’s publicly funded safety net health system predominantly serves uninsured patients and those with Medicaid, a federal and state government-funded program that provides coverage to eligible low-income U.S. citizens, nationals, and certain immigrant residents meeting strict criteria [16]. In Harris County, county-funded plans are available for certain undocumented immigrants living below the federal poverty line. Individuals who are not eligible for a coverage plan can use health system services by paying out-of-pocket (i.e., self-pay). For additional information about health insurance plans see Supplementary Table 1.

### Participants

Our study included women who were aged 30–65 years and attended for primary care in the safety net health system at least twice between March 1, 2015, and February 28, 2020. Patients with history of hysterectomy, cervical cancer, or cervical dysplasia in the five years prior were excluded.

### Data collection

Data were retrieved from a query of the EHR database as part of an ongoing randomized clinical trial to evaluate self-sample HPV testing as a strategy to increase screening coverage [17].

### Measures

The main outcome of screening was determined based on the current USPSTF cervical cancer screening guidelines. Patients aged 30–65 years were considered UTD on screening if they had a Pap test in the past 3.5 years or co-test or HPV test in the past 5.5 years. A 6-month grace period was included beyond the intervals in the guidelines to allow time for women to respond to opportunistic usual care strategies (i.e., in-clinic EHR-flagging and video-based patient education). The main independent variable of interest was preferred language among Hispanic women (i.e., English or Spanish). Other potential confounders that were assessed include age (years), tobacco use (former, current, never), and insurance coverage (private, indigent/county, Medicaid, Medicare, none/self-pay, other).

### Analysis

Data were prepared and managed in Microsoft Excel and imported into SAS version 9.4 for all analyses (SAS Institute Cary, NC). Descriptive statistics were generated, and chi-square tests for independence were conducted to assess the relationships between categorical independent variables and UTD screening, while the t-test with unequal variance was conducted to assess the relationship between age and UTD screening. Additionally, the ANOVA test was utilized to assess the difference in mean age across racial/ethnic groups. Univariate and multivariable log-binomial regression models were generated using the PROC GENMOD in SAS which produced unadjusted prevalence ratios (PR), adjusted prevalence ratios (aPR), and 95% confidence intervals (CI) to assess the association between UTD screening and independent variables. All statistical tests were two-tailed with an alpha probability of 0.05.

This study was reviewed and approved by the Institutional Review Boards for Baylor College of Medicine and Affiliated Hospitals prior to data abstraction. The requirement to obtain informed consent/HIPA authorization for this research was waived by the Institutional Review Board for Baylor College of Medicine and Affiliated Hospitals.

## Results

A total of 47,595 women were seen within the health system between March 2015 and February 2020, and 46,094 women had complete data for race/ethnicity and screening status. Of these, most women were Hispanic (67.9%). As shown in Table 1, the average age of Hispanic women overall was 46 years, the majority were Spanish-speaking (83.9%), almost half had indigent/county healthcare plans (48.8%) and 22.6% had no insurance, 86.6% never smoked tobacco, and 81.5% were UTD on screening which was higher than that of non-Hispanic Black (74.7%) and White (78.1%) women. Specific pairwise comparisons are available in Supplementary Table 2.

**Table 1:** Characteristics of the safety net population stratified by race/ethnicity, *N* = 46,094

Characteristics	Race/ethnicity				*p*-value
	Hispanic	Black	White	Other	
	*n* = 31,297 (67.9)	*n* = 9207 (20.0)	*n* = 3026 (6.6)	*n* = 2564 (5.6)	
	n (col %)	n (col %)	n (col %)	n (col %)	
Age, years (mean, SD)	46.0, 9.5	47.7, 10.4	48.8, 10.3	50.5, 10.1	** < 0.0001**
Preferred language					** < 0.0001**
* English*	4983 (15.9)	9030 (98.1)	2657 (87.8)	1040 (40.6)	
* Spanish*	26,269 (83.9)	26 (0.3)	323 (10.7)	6 (0.2)	
* Other*	45 (0.1)	151 (1.6)	46 (1.5)	1518 (59.2)	
Insurance					** < 0.0001**
* Private*	5977 (19.1)	2403 (26.1)	710 (23.5)	1221 (47.6)	
* Indigent/County*	15,260 (48.8)	2352 (25.6)	956 (31.6)	936 (36.5)	
* Medicaid*	2150 (6.9)	1840 (20.0)	533 (17.6)	171 (6.7)	
* Medicare*	432 (1.4)	907 (9.9)	280 (9.3)	39 (1.5)	
* No insurance/self-pay*	7086 (22.6)	1455 (15.8)	441 (14.6)	194 (7.6)	
* Other*	392 (1.3)	250 (2.7)	106 (3.5)	3 (0.1)	
Tobacco use					** < 0.0001**
* Current*	1500 (4.8)	2147 (23.4)	990 (32.8)	64 (2.5)	
* Former*	2686 (8.6)	1384 (15.1)	702 (23.2)	66 (2.6)	
* Never*	27,062 (86.6)	5645 (61.5)	1330 (44.0)	2429 (94.9)	
Screening status					** < 0.0001**
* Not UTD*	5779 (18.5)	2330 (25.3)	664 (21.9)	264 (10.3)	
* UTD*	25,518 (81.5)	6877 (74.7)	2362 (78.1)	2300 (89.7)	

*Abbreviations SD* Standard deviation, *UTD* Up to date

Table 2 displays the cross-tabulations of independent variables by screening status in English- and Spanish-speaking Hispanic women. Spanish-speaking Hispanic women (82.7%) had a higher proportion of those UTD than English speakers (75.4%). By insurance type, those with county healthcare plans had the highest proportion of those UTD (89.5%). UTD status was also associated with tobacco use. Specific pairwise comparisons are available in Supplementary Table 3.

**Table 2:** Selected characteristics by cervical cancer screening status among English- and Spanish-speaking Hispanic women, *N* = 31,252

	Screening status		*p*-value
	UTD	Not UTD	
	n (row %)	n (row %)	
Age, years (mean, SD)	46.1, 9.4	45.8, 10.1	0.0538
Language-use group			** < 0.0001**
* English-speaking Hispanic*	3755 (75.4)	1228 (24.6)	
* Spanish-speaking Hispanic*	21,724 (82.7)	4545 (17.3)	
Insurance			** < 0.0001**
* Private*	4803 (80.5)	1163 (19.5)	
* Indigent/County*	13,641 (89.5)	1597 (10.5)	
* Medicaid*	1445 (67.3)	702 (32.7)	
* Medicare*	319 (74.4)	110 (25.6)	
* No insurance/self-pay*	4986 (70.4)	2094 (29.6)	
* Other*	285 (72.7)	107 (27.3)	
Tobacco use			**0.0013**
* Current*	1208 (80.6)	291 (19.4)	
* Former*	2122 (79.1)	561 (20.9)	
* Never*	22,117 (81.9)	4904 (18.2)	

*Abbreviations SD* Standard deviation, *UTD* Up to date

Table 3 shows the unadjusted and adjusted prevalence ratios assessing the relationship between screening status and independent variables in English- and Spanish-speaking Hispanic women. In multivariable analyses, English-speaking Hispanic women had a lower prevalence of UTD screening when compared to Spanish-speaking Hispanic women (aPR: 0.94, 95% CI: 0.93 – 0.96). Additionally, those with Medicaid (aPR: 0.84, 95% CI: 0.82 – 0.87), Medicare (aPR: 0.94, 95% CI: 0.89 – 0.99), no insurance/self-pay (aPR: 0.87, 95% CI: 0.85 – 0.89), or other insurance (aPR: 0.90, 95% CI: 0.85 – 0.96) had a lower prevalence of UTD screening when compared to those with private insurance, while those with indigent/county healthcare plans had a higher prevalence of UTD screening when compared to those with private insurance (aPR: 1.10, 95% CI: 1.09 – 1.12).

**Table 3:** Univariate and multivariable associations between selected characteristics and cervical cancer screening status

	UTD on Screening			
	PR (95% CI)	*p*-value	aPR (95% CI)	*p*-value
Age, years	**1.00 (1.00—1.001)**	**0.06**	**1.00 (1.00–1.001)**	**0.60**
Language				
* Spanish-speaking Hispanic*	1.00		1.00	
* English-speaking Hispanic*	**0.91 (0.90 – 0.93)**	** < 0.0001**	**0.94 (0.93 – 0.96)**	** < 0.0001**
Insurance				
* Private*	1.00		1.00	
* Indigent/County*	**1.11 (1.10 – 1.13)**	** < 0.0001**	**1.10 (1.09 – 1.12)**	** < 0.0001**
* Medicaid*	**0.84 (0.81 – 0.86)**	** < 0.0001**	**0.84 (0.82 – 0.87)**	** < 0.0001**
* Medicare*	**0.92 (0.87 – 0.98)**	**0.0063**	**0.94 (0.89 – 0.99)**	**0.02**
* No insurance/self-pay*	**0.87 (0.86 – 0.89)**	** < 0.0001**	**0.87 (0.85 – 0.89)**	** < 0.0001**
* Other*	**0.90 (0.85 – 0.96)**	**0.0013**	**0.90 (0.85 – 0.96)**	**0.0008**
Tobacco use				
* Never*	1.00		1.00	
* Current*	0.98 (0.96 – 1.01)	0.23	1.00 (0.98 – 1.03)	0.78
* Former*	**0.97 (0.95 – 0.99)**	**0.0009**	**0.98 (0.96 – 1.00)**	**0.03**

*Abbreviations*: *UTD* Up to date, *PR* Prevalence ratio, *CI* Confidence interval, *aPR* Adjusted prevalence ratio, *SD* Standard deviation

## Discussion

While Hispanic women are often considered a homogenous group in the literature, the findings from our study suggest significant differences within the Hispanic population that may not regularly be captured within the standard race/ethnicity categories typically used to abstract demographics and/or categorize subjects. By examining patterns of care among Hispanic women stratified according to their preferred language, our results indicate that Spanish-speaking women in an urban safety net system are more screening-adherent when compared to English-speaking Hispanic women. Our findings, reflective of a public safety net health system that provides care to a predominantly uninsured, racial/ethnic minority population, are contrary to what is generally reported for the broader Hispanic population, with higher screening rates among English speakers [9]. We hypothesize that because all women in the safety net health system have access to healthcare services, it is possible that nativity-related disparities, observed in the broader population, are addressed by removing barriers related to inadequate healthcare access. In this health system, primarily Spanish-speaking Hispanic women were more likely to access care via county healthcare programs, rather than Medicaid, as they would not otherwise qualify for Medicaid due to their citizenship status. Interestingly, we found that Hispanic women with indigent/county healthcare plans had higher UTD screening when compared to all other types of health insurance coverage. We suspect that county healthcare plans may circumvent certain barriers potentially encountered by patients with private insurance, Medicaid, and self-pay, including co-pays for clinical visits and fragmentation of care across multiple healthcare organizations. Further research is needed to elucidate the reasons for these findings.

We also found that Hispanic women who were former but not current smokers had slightly lower UTD screening compared to never-smokers, which is contrary to previous literature. However, several factors associated with smoking status that we were unable to adjust for, such as comorbidities and frequency of smoking, may be affecting screening adherence [18, 19]. More research may be warranted to help clarify the relationship between smoking behavior and screening adherence.

Our findings should be interpreted considering certain limitations. First, cross-sectional data do not allow for the study of causality. Second, limited variables were available in the EHR query, thus we were not able to assess all potentially important covariates, including income and education. Third, our findings primarily relate to Hispanics living in the U.S. as well as the U.S. safety net healthcare system. Given that healthcare programs are different across countries, our results may not be completely generalizable in non-U.S. healthcare settings. However, while every country’s healthcare system is different, we believe our study identifies potential opportunities to decrease disparities in the Spanish-speaking Hispanic population regardless of country as well as underscores the need to assess populations on a disaggregate level, as aggregate data often mask disparities [20]. Further, our study highlights considerations for immigrant health care in general, not limited to the Hispanic population, as health inequity, specifically related to cervical cancer screening, has been observed internationally in non-Hispanic migrant women [21, 22].

## Conclusions

The findings from this paper highlight differences in cervical cancer screening adherence in Hispanic subpopulations within an urban safety net healthcare system among Spanish- and English-speaking Hispanic patients. The Hispanic population is heterogeneous and should be disaggregated in research to appropriately inform effective interventions aimed at decreasing health disparities within this population. Removing access to health insurance-related barriers may be an important facilitator for improving cervical cancer screening among Spanish-speaking Hispanic women. Further, our findings hold implications for providing strategic and targeted programs to increase cervical cancer screening rates among those with lower rates of screening.

## Supplementary Information

**Additional file 1: **Supplementary materials. **Supplementary Table 1.** Description of Terms. **Supplementary Table 2.** Post-hoc analyses assessing pairwise comparisons of omnibus testing presented in Table 1. **Supplementary Table 3.** Post-hoc analyses assessing pairwise comparisons of omnibus testing presented in Table 2. 

## Data Availability

The datasets generated and analyzed during the current study are not publicly available due to the data being a part of a query of private electronic health records as part of the larger clinical trial.
