# Supplementary material for: Cervical cancer screening among English- and Spanish-speaking Hispanic women in an urban safety net health system, 2015–2020
Source: BMC Womens Health. 2023 Jun 15;23:309. doi: 10.1186/s12905-023-02448-3 (PMC10268437; doi:10.1186/s12905-023-02448-3)
Supplement: Supplementary file 1 — Additional file 1: Supplementary materials. Supplementary Table 1. Description of Terms. Supplementary Table 2. Post-hoc analyses assessing pairwise comparisons of omnibus testing presented in Table 1. Supplementary Table 3. Post-hoc analyses assessing pairwise comparisons of omnibus testing presented in Table 2. [file 12905_2023_2448_MOESM1_ESM.pdf]

SUPPLEMENTARY MATERIALS

Cervical cancer screening among English- and Spanish-speaking Hispanic women in an urban safety net health system, 2015-2020

Trisha L. Amboree [1,2], Susan Lackey Parker [1,2,3], Shaun Bulsara [3], Matthew L. Anderson [4], Kathleen M. Schmeler [5], Elizabeth Y. Chiao [6], Jane R. Montealegre [1,2,3]

[1] The University of Texas MD Anderson Cancer Center, Department of Behavioral Science, Houston, Texas, USA

[2] Baylor College of Medicine, Department of Pediatrics, Houston, Texas, USA

[3] Dan L. Duncan Comprehensive Cancer Center, Baylor College of Medicine, Houston, Texas, USA

[4] University of South Florida Morsani School of Medicine and Tampa General Hospital Cancer Institute, Division of Gynecologic Oncology, Tampa, Florida, USA

[5] The University of Texas MD Anderson Cancer Center, Department of Gynecologic Oncology and Reproductive Medicine, Houston, Texas, USA

[6] The University of Texas MD Anderson Cancer Center, Departments of Epidemiology and Medical Oncology, Houston, Texas, USA

Corresponding author:
Trisha L. Amboree, PhD, MPH
The University of Texas MD Anderson Cancer Center
1515 Holcombe Blvd., Unit 1330, Houston, TX 77030
Tel: 713- 563-6079
Email: TLAmboree@mdanderson.org

Supplementary Table 1. Description of Terms

	Description
Safety net health system	United States (U.S.) healthcare settings that provide health care to vulnerable populations regardless of their ability to pay or their insurance coverage. The specific safety net referenced in this study provides care to a largely uninsured population made up of mostly non-White individuals (i.e., Hispanic, African American, Asian, and other).
Private insurance	Also called commercial health insurance, this is health coverage that is issued by a private U.S. company or entity (i.e., non-government company) to U.S. nationals, citizens, permanent residents, or legal aliens.
Indigent/county insurance	Health insurance plans within the safety net health system that are available to low-income county residents. The cost to the patient is based on their annual household income (i.e., if income is higher, the cost may be higher and vice versa). These health plans are also provided to patients regardless of immigration status (i.e., regardless of U.S. resident status).
Medicaid	U.S. government health insurance coverage offered to low-income or very low-income U.S. nationals, citizens, permanent residents, or legal aliens, who are also legal residents of their state and need health insurance assistance.
Medicare	U.S. government health insurance coverage offered to low-income U.S. nationals, citizens, permanent residents, or legal aliens, who are aged 65 years or older and need health insurance assistance.
No insurance/self-pay	Patients with no health insurance coverage who are required to pay for their medical expenses out-of-pocket.

References:

1. Topic: Safety Net. Agency for Healthcare Research and Quality. U.S. Department of Health and Human Services. Accessed April 6, 2023. https://www.ahrq.gov/topics/safety-net.html#:~:text=Safety%20net%20practices%20are%20defined%20by%20the%20Institute,services%20to%20uninsured%2C%20Medicaid%20and%20other%20vulnerable%20patients.%22

2. Texas Health Insurance. Texas.gov. Texas Department of Insurance. Accessed April 10, 2023. https://www.texas.gov/health-services/texas-health-insurance/

3. County Indigent Health Care Program. Texas Health and Human Services. Accessed April 10, 2023. https://www.hhs.texas.gov/services/health/county-indigent-health-care-program#:~:text=The%20County%20Indigent%20Health%20Care,preventative%20and%20some%20specialty%20services.

Supplementary Table 2. Post-hoc analyses assessing pairwise comparisons of omnibus testing presented in Table 1

	Race/ethnic group											
	White vs. Hispanic		White vs. Black		White vs. Other		Hispanic vs. Black		Hispanic vs. Other		Black vs. Other	
Characteristics	Raw p-value	Adj. p-value	Raw p-value	Adj. p-value	Raw p-value	Adj. p-value	Raw p-value	Adj. p-value	Raw p-value	Adj. p-value	Raw p-value	Adj. p-value
Age, years (mean, SD)	<.0001	<.0001	0.0002	0.0011	<.0001	<.0001	<.0001	<.0001	<.0001	<.0001	<.0001	<.0001
Preferred language												
English vs. Spanish	<.0001	<.0001	<.0001	<.0001	<.0001	<.0001	<.0001	<.0001	<.0001	<.0001	0.1185	0.7107
English vs. Other	0.0017	0.0103	0.8380	1.000	<.0001	<.0001	0.0002	0.0015	<.0001	<.0001	<.0001	<.0001
Spanish vs. Other	<.0001	<.0001	<.0001	<.0001	<.0001	<.0001	<.0001	<.0001	<.0001	<.0001	<.0001	<.0001
Insurance												
Private vs. Indigent/County	<.0001	<.0001	<.0001	<.0001	<.0001	<.0001	<.0001	<.0001	<.0001	<.0001	<.0001	<.0001
Private vs. Medicaid	<.0001	<.0001	0.7613	1.0000	<.0001	<.0001	<.0001	<.0001	<.0001	<.0001	<.0001	<.0001
Private vs. Medicare	<.0001	<.0001	0.5864	1.0000	<.0001	<.0001	<.0001	<.0001	<.0001	<.0001	<.0001	<.0001
Private vs. No insurance/self-pay	<.0001	<.0001	0.7123	1.0000	<.0001	<.0001	<.0001	<.0001	<.0001	<.0001	<.0001	<.0001
Private vs. Other	<.0001	<.0001	0.0033	0.0199	<.0001	<.0001	<.0001	<.0001	<.0001	<.0001	<.0001	<.0001
Indigent/County vs. Medicaid	<.0001	<.0001	<.0001	<.0001	<.0001	<.0001	<.0001	<.0001	0.0025	0.0152	<.0001	<.0001
Indigent/County vs. Medicare	<.0001	<.0001	0.0004	0.0026	<.0001	<.0001	<.0001	<.0001	0.0226	0.1357	<.0001	<.0001
Indigent/County vs. No insurance/self-pay	0.9114	1.0000	<.0001	<.0001	<.0001	<.0001	<.0001	<.0001	<.0001	<.0001	<.0001	<.0001
Indigent/County vs. Other	<.0001	<.0001	0.7293	1.0000	<.0001	<.0001	<.0001	<.0001	<.0001	0.0001	<.0001	<.0001
Medicaid vs. Medicare	<.0001	<.0001	0.4498	1.0000	<.0001	<.0001	<.0001	<.0001	0.4935	1.0000	<.0001	<.0001
Medicaid vs. No insurance/self-pay	<.0001	<.0001	0.5368	1.0000	0.0102	0.0611	<.0001	<.0001	<.0001	<.0001	0.0011	0.0063
Medicaid vs. Other	0.4678	1.0000	0.0024	0.0142	<.0001	<.0001	0.0007	0.0042	<.0001	<.0001	<.0001	0.0002
Medicare vs. No insurance/self-pay	<.0001	<.0001	0.8335	1.0000	<.0001	<.0001	<.0001	<.0001	<.0001	<.0001	<.0001	<.0001

Medicare vs. Other	<.0001	<.0001	0.0181	0.1085	0.0041	0.0246	<.0001	<.0001	<.0001	<.0001	0.0240	0.1441
No insurance/self-pay vs. Other	<.0001	<.0001	0.0085	0.0512	<.0001	<.0001	<.0001	<.0001	0.0197	0.1182	<.0001	<.0001
Tobacco use												
Current vs. Former	<.0001	<.0001	0.1132	0.6793	0.0389	0.2336	<.0001	<.0001	0.0018	0.0105	0.0081	0.0483
Current vs. Never	<.0001	<.0001	<.0001	<.0001	<.0001	<.0001	<.0001	<.0001	<.0001	<.0001	<.0001	<.0001
Former vs. Never	<.0001	<.0001	<.0001	<.0001	<.0001	<.0001	<.0001	<.0001	<.0001	<.0001	<.0001	<.0001
Screening status												
UTD vs. Not UTD	<.0001	<.0001	0.0002	0.0011	<.0001	<.0001	<.0001	<.0001	<.0001	<.0001	<.0001	<.0001
Raw p-value is based on post-hoc pairwise comparison using chi-square testing.<br>Adjusted p-value is based on Bonferroni correction.												

Supplementary Table 3. Post-hoc analyses assessing pairwise comparisons of omnibus testing presented in Table 2.

	Screening status	
	UTD vs. Not UTD	
Characteristic	Raw p-value	Adj. p-value
Age, years (mean, SD)	0.0538	---
Language-use group		
English- vs. Spanish-speaking Hispanic	<.0001	<.0001
Insurance		
Private vs. Indigent/County	<.0001	<.0001
Private vs. Medicaid	<.0001	<.0001
Private vs. Medicare	0.0021	0.0311
Private vs. No insurance/self-pay	<.0001	<.0001
Private vs. Other	0.0002	0.0027
Indigent/County vs. Medicaid	<.0001	<.0001
Indigent/County vs. Medicare	<.0001	<.0001
Indigent/County vs. No insurance/self-pay	<.0001	<.0001
Indigent/County vs. Other	<.0001	<.0001
Medicaid vs. Medicare	0.0041	0.0612
Medicaid vs. No insurance/self-pay	0.0059	0.0878
Medicaid vs. Other	0.0348	0.5224
Medicare vs. No insurance/self-pay	0.0822	1.0000
Medicare vs. Other	0.5912	1.0000
No insurance/self-pay vs. Other	0.3350	1.0000
Tobacco use		
Current vs. Former	0.2492	0.7477
Current vs. Never	0.2171	0.6513
Former vs. Never	0.0004	0.0013
Raw p-value is based on post-hoc pairwise comparison using chi-square testing. Adjusted p-value is based on Bonferroni correction.		
